# Synthesis of nanoparticle CT contrast agents: *in vitro* and *in vivo* studies

**DOI:** 10.1088/1468-6996/16/5/055003

**Published:** 2015-10-09

**Authors:** Sung June Kim, Wenlong Xu, Md Wasi Ahmad, Jong Su Baeck, Yongmin Chang, Ji Eun Bae, Kwon Seok Chae, Tae Jeong Kim, Ji Ae Park, Gang Ho Lee

**Affiliations:** 1 Department of Chemistry, College of Natural Sciences, Kyungpook National University (KNU), Taegu 702-701, Korea; 2 Department of Molecular Medicine and Medical & Biological Engineering, School of Medicine, KNU and Hospital, Taegu 702-701, Korea; 3 Department of Nanoscience and Nanotechnology, KNU, Taegu 702-701, Korea; 4 Department of Biology Education, Teacher’s College, KNU, Taegu 702-701, Korea; 5 Institute of Biomedical Engineering Research, KNU, Taegu 702-701, Korea; 6 Laboratory of Nuclear Medicine Research, Molecular Imaging Research Center, Korea Institute of Radiological Medical Science, Nowon-gil 75, Seoul 139-706, Korea

**Keywords:** nanoparticle, Na_2_WO_4_, BaCO_3_, CT contrast agent

## Abstract

Water-soluble and biocompatible D-glucuronic acid coated Na_2_WO_4_ and BaCO_3_ nanoparticles were synthesized for the first time to be used as x-ray computed tomography (CT) contrast agents. Their average particle diameters were 3.2 ± 0.1 and 2.8 ± 0.1 nm for D-glucuronic acid coated Na_2_WO_4_ and BaCO_3_ nanoparticles, respectively. All the nanoparticles exhibited a strong x-ray attenuation. *In vivo* CT images were obtained after intravenous injection of an aqueous sample suspension of D-glucuronic acid coated Na_2_WO_4_ nanoparticles, and positive contrast enhancements in the kidney were clearly shown. These findings indicate that the nanoparticles reported in this study may be promising CT contrast agents.

## 1. Introduction

Molecular imaging is a vital tool for the diagnosis of diseases such as cancers [1–4]. Among various imaging modalities, x-ray computed tomography (CT) is a very useful technique which allows bones and hardened diseases such as cancers to be imaged because soft tissues are almost transparent to x-ray beams [5]. With CT contrast agents, however, soft tissues and blood vessels can be also imaged [6–9], allowing for various parts of the body including bones and soft tissues to be imaged using CT contrast agents, including cancers [6, 8–10].

Until recently tri-iodinated organic molecules functionalized with hydrophilic groups for water-solubility have been used as injectable CT contrast agents in clinical practice. These include Ultravist^®^, Omnipaque™, Visipaque™, etc [7–9]. In these molecules iodines attenuate (absorb and scatter) the x-ray beam, thereby acting as CT contrast agents [7–9]. Iodine contrast agents can be highly concentrated in iodine (1.0–2.5 M [I]), because there are three iodines per monomer-molecule and six iodines per dimer-molecule [10]. To provide adequate contrast, large doses of iodine contrast agents are generally administered; however, these high doses might have side-effects in patients [11]. Injection doses of CT contrast agents in terms of number density can be reduced by the use of nanoparticles because at the same atomic concentration, the number density of the nanoparticles is much lower than the number densities of molecular agents. Because both viscosity and osmolarity are very low for nanoparticle agents, higher atomic concentrations can be injected by slightly increasing the number density of nanoparticle agents to obtain higher contrasts [12]. Because of the large linear x-ray attenuation coefficients^7^ 7Linear x-ray attenuation coefficients (*μ*) can be calculated by multiplying x-ray mass attenuation coefficient (*μ*/*ρ*) (see [13]) by density (*ρ*) and are directly available at http://dwb.unl.edu/teacher/nsf/c04/c04links/www.csrri.iit.edu/periodic-table.html. (*μ*) of heavy metals (generally larger than iodine) [13], the injection doses of the heavy metal-containing nanoparticles can be also further reduced [12].

Despite the above-mentioned advantages of metal-containing nanoparticles over molecular agents, only a few nanoparticle CT contrast agents have been studied thus far [12, 14–20]. These include gold (Au) nanoparticles [12, 14–16], Gd(IO_3_)_3_ nanoparticles [17], Gd_2_O_3_ nanoparticles [19, 20], and tantalum oxide nanoparticles [21], and bismuth sulphide nanoparticles [22]. It is important to note that gold, tantalum, and bismuth have much larger linear x-ray attenuation coefficients than iodine, as does Gd [13], (see footnote 7). Tungsten (W) and barium (Ba) which are studied in this work, also have large linear x-ray attenuation coefficients such that W ≫ I ≈ Ba at an x-ray source voltage ofsss 70 keV [13], (see footnote 7). These large linear x-ray attenuation coefficients of metals as well as the large amount of metal per nanoparticle certainly make these metal-containing nanoparticles promising CT contrast agents worthy of further study.

In this study, two kinds of metal-containing nanoparticles, namely D-glucuronic acid coated Na_2_WO_4_ and BaCO_3_ nanoparticles were synthesized for the first time and their CT imaging properties were studied *in vitro* and *in vivo*: x-ray attenuation phantom images were recorded and *in vivo* CT images were obtained after intravenous injection of an aqueous sample suspension of D-glucuronic acid coated Na_2_WO_4_ nanoparticles into the tail vein of a mouse. These findings of this study indicate that the metal-containing nanoparticles may be promising CT contrast agents.

## 2. Experimental details

### 2.1. Synthesis

Tungsten chloride (WCl_6_, 99.9+%), barium chloride dihydrate (BaCl_2_ · 2H_2_O, ≥99%), triethylene glycol (99%, boiling point = 285 °C), D-glucuronic acid (99%), and NaOH (≥98%) were purchased from Sigma-Aldrich, USA and used as received. Ethanol (99.9%) was purchased from Duksan Chemical, South Korea and used to wash the nanoparticles, while triply distilled water was used for the final wash of the nanoparticles and in the preparation of aqueous sample suspensions.

**Figure 1.**
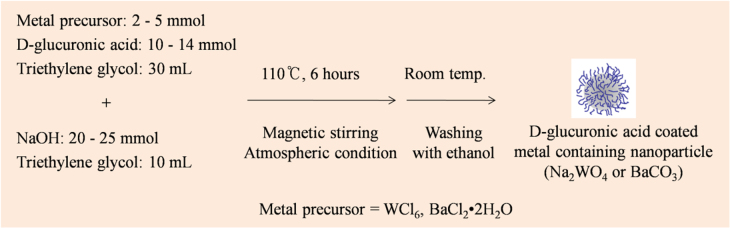
A reaction scheme used for the one-pot synthesis of D-glucuronic acid coated Na_2_WO_4_ and BaCO_3_ nanoparticles. The exact amounts of chemicals used in the synthesis are described in the text.

All samples were synthesized using the one-pot synthesis scheme (figure 1). To synthesize D-glucuronic acid coated Na_2_WO_4_ nanoparticles, two separate solutions were prepared: (i) a precursor solution made of 2 mmol of precursor and 14 mmol of D-glucuronic acid in 30 mL of triethylene glycol; and (ii) a NaOH solution with 25 mmol of NaOH in 10 mL of triethylene glycol. The precursor solution was magnetically stirred at 60 °C under atmospheric conditions until the precursor was completely dissolved in the solvent, after which the NaOH solution was added to the precursor solution. The mixed solution was magnetically stirred at 110 °C for 6 h. To synthesize D-glucuronic acid coated BaCO_3_ nanoparticles, 5 mmol of BaCl_2_ · 2H_2_O, 10 mmol of D-glucuronic acid, and 20 mmol of NaOH were used. The reaction procedures and conditions were the same as those used for D-glucuronic acid coated Na_2_WO_4_ nanoparticles. To wash the product samples with ethanol, the solution was cooled to room temperature and transferred to a 1 L beaker containing 500 mL ethanol. It was then magnetically stirred for 10 min and stored for a week to let the nanoparticle samples settle to the bottom of the beaker. The clear supernatant was decanted and the remaining sample was again diluted with 500 mL ethanol. This washing process was repeated three times. Half the volume of each sample was dried in air to obtain a powder sample for characterization, and the remaining half of each sample was washed three more times with triply distilled water to prepare an aqueous sample suspension. Typical yields of the product nanoparticles were ∼60%.

### 2.2. Characterization

The diameters of synthesized nanoparticles were measured using a high resolution transmission electron microscope (HRTEM) (FEI, Titan G2 ChemiSTEM CS Probe) operated at an acceleration voltage of 200 kV. For each sample, one drop of the nanoparticle sample dispersed in ethanol was dropped onto a carbon film supported by a 200 mesh copper grid (PELCO No.160, TED PELLA, INC.) placed on a filter paper using a micropipette (Eppendorf, 2–20 L). The copper grid with the sample was left in air to dry for an hour at room temperature, after which it was mounted inside the HRTEM for measurements.

The metal concentration in each sample suspension prepared in triply distilled water was determined using an inductively coupled plasma atomic emission spectrometer (Thermo Jarrell Ash Co., IRIS/AP). All samples were pre-treated with acids to completely dissolve the nanoparticles in solution before measurements were taken.

The hydrodynamic diameters of the nanoparticles dispersed in triply distilled water were measured using a dynamic light scattering (DLS) particle size analyzer (UPA-150, Microtrac). The sample solution concentration used for measurements was ∼0.05 mM [W] or [Ba].

The crystal structure of the powder samples before and after thermo-gravimetric analysis (TGA) was measured using a powder x-ray diffraction (XRD) spectrometer (Philips, X-PERT PRO MRD) with unfiltered CuKα (*λ* = 1.541 84 Å) radiation. The scanning step and scan range in 2*θ* were 0.033° and 15°–100°, respectively.

The surface coating of the nanoparticles with D-glucuronic acid was investigated by recording Fourier transform infrared (FT-IR) absorption spectra of the powder samples using an FT-IR absorption spectrometer (Mattson Instruments, Inc., Galaxy 7020A). For the measurements, the powder samples were dried on a hot plate at ∼40 °C for a week. Pellets of the dried powder samples dispersed in KBr were prepared, and FT-IR absorption spectra were recorded between 400 and 4000 cm^−1^.

The number of D-glucuronic acid molecules coating the nanoparticle surfaces was estimated by recording TGA curves of the powder samples generated using a TGA instrument (TA Instruments, SDT-Q 600). As the organic compounds mostly burn out below 400 °C, TGA curves were scanned between room temperature and 900 °C under air flow. The surface coating in each sample was quantified from the mass drop in the TGA curve after taking into account the initial mass drop due to water desorption between room temperature and ∼105 °C.

The cellular toxicities of the sample suspensions prepared in triply distilled water were assessed using the CellTiter-Glo Luminescent Cell Viability Assay (Promega, Wisconsin, USA). In this assay, intracellular adenosine triphosphate was quantified using a luminometer (Victor 3, Perkin-Elmer). Human prostate cancer (DU145) and normal mouse hepatocyte (NCTC1469) cell lines were used. The cells were seeded onto 24-well cell culture plates and incubated for 24 h (5 × 10^4^ cells/well, 500 *μ*L cell suspension per well, 5% CO_2_, and 37 °C). DMEM and RPMI1640 were used as culture media for NCTC1469 and DU145 cells, respectively. Four test suspensions (10, 50, 100, and 200 *μ*M [W] or [Ba]) were prepared by diluting an original sample suspension with a sterile phosphate-buffered saline solution. 2 *μ*L of each test sample was added to the cultured cells, which were then incubated for a further 48 h. The viabilities of the treated cells in each well were measured and normalized to the viabilities of untreated control cells. These measurements were repeated twice for each well to obtain average cell viabilities.

X-ray phantom images were acquired using a micro-CT scanner (Siemens, Inveon). Two dilute solutions per sample suspension (100 and 150 mM [W] for D-glucuronic acid coated Na_2_WO_4_ nanoparticles; 100 and 155 mM [Ba] for D-glucuronic acid coated BaCO_3_ nanoparticles) were prepared. A phantom image of water was also measured as a reference for Hounsfield units (HU) of 0.0. Phantom images of a commercial iodine CT contrast agent (Ultravist^®^; 56, 100, and 150 mM [I]) were also measured for comparison. The x-ray attenuation of each sample suspension was estimated in HU with respect to water. The parameters used for measurements were as follows: the x-ray source current = 100 *μ*A, the x-ray source voltage = 70 kV, the imaging time per frame = 200 ms, and the reconstructed image size = 512 × 512.

*In vivo* CT images of a mouse were acquired using the same micro-CT scanner used for the phantom image measurements. All animal experiments were approved by the animal research committee of Kyungpook National University and conducted in accordance with its rules. A female ICR mouse (∼40 g) was used for the measurements, and the injection dose was typically ∼0.015 mmol W kg^−1^. For imaging, the mouse was anesthetized using 1.5% isoflurane in oxygen, and measurements were made before and after injection of a sample suspension into the tail vein. After the measurement, the mouse was revived from anaesthesia and placed in a cage with free access to food and water. The parameters used for measurements were as follows: the x-ray source current = 400 *μ*A, the x-ray source voltage = 70 kV, the imaging time per frame = 200 ms, and the reconstructed image size = 512 × 512.

## 3. Results and discussion

### 3.1. Particle diameter, hydrodynamic diameter, and crystal structure

**Figure 2.**
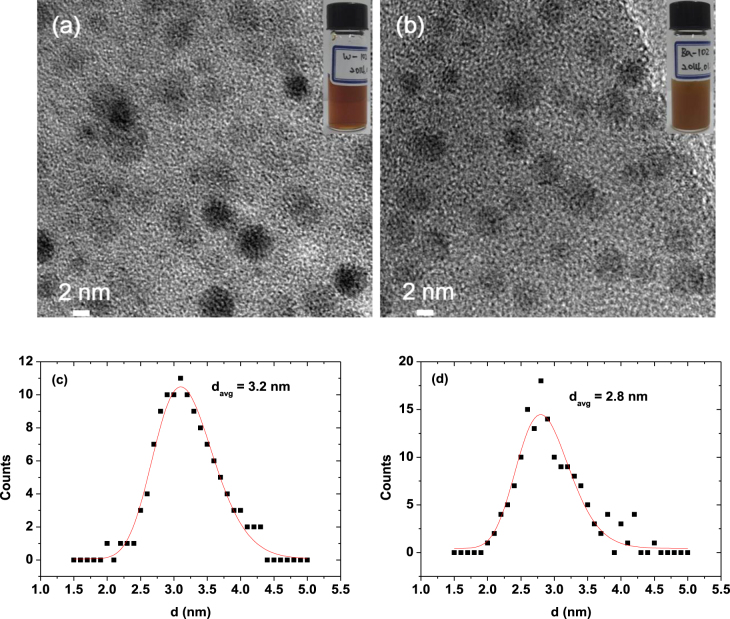
HRTEM images and photographs (inserts) of aqueous sample suspensions: D-glucuronic acid coated (a) Na_2_WO_4_ and (b) BaCO_3_ nanoparticles. The corresponding particle diameter distributions fitted with log-normal functions are provided in (c) and (d), respectively.

All nanoparticles synthesized in this study had small diameters (figures 2(a) and (b)). The average particle diameters were estimated to be 3.2 ± 0.1 nm for D-glucuronic acid coated Na_2_WO_4_ nanoparticles, and 2.8 ± 0.1 nm for D-glucuronic acid coated BaCO_3_ nanoparticles from the log-normal function fitting of the particle diameter distributions (figures 2(c), (d) and table 1). A representative photograph of each aqueous sample suspension is inserted in the respective HRTEM image (figure 2), showing that good colloidal suspensions were achieved for both samples. We propose that Na_2_WO_4_ and BaCO_3_ nanoparticles were produced by the following reactions, respectively

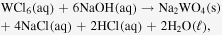


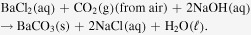



**Table 1.** Average particle diameter (*d*_avg_), average hydrodynamic diameter (*a*_avg_), and surface coating amount (*P*, *σ*, *N*) of D-glucuronic acid coated Na_2_WO_4_ and BaCO_3_ nanoparticles.

				Surface coating amount		
Nanoparticle	Ligand	*d*_avg_ (nm)	*a*_avg_ (nm)	*P* (%)	*σ* (molecules/nm^2^)	*N* (molecules/nanoparticle)
Na_2_WO_4_	D-glucuronic acid	3.2 ± 0.1	8.9 ± 0.1	54 ± 1	11.0	1328
BaCO_3_	D-glucuronic acid	2.8 ± 0.1	8.2 ± 0.1	40 ± 1	5.1	502

**Figure 3.**
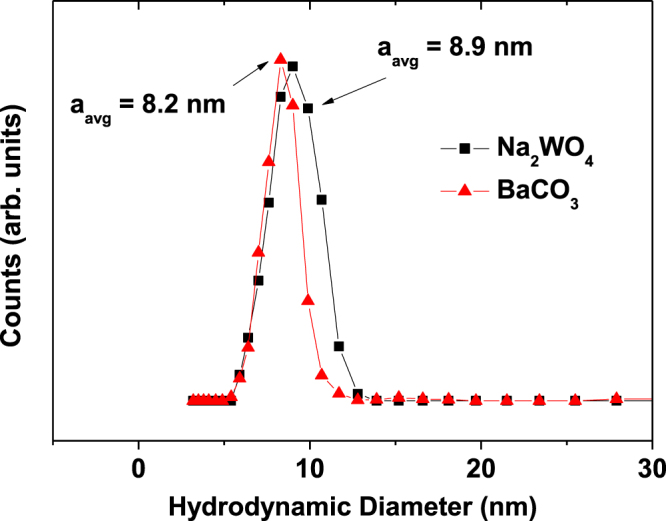
DLS patterns of aqueous sample suspensions of D-glucuronic acid coated Na_2_WO_4_ (■) and BaCO_3_ (▲) nanoparticles.

The DLS patterns in the aqueous sample suspensions were measured (figure 3), and the average hydrodynamic diameters were estimated to be 8.9 ± 0.1 nm for D-glucuronic acid coated Na_2_WO_4_ nanoparticles and 8.2 ± 0.1 nm for D-glucuronic acid coated BaCO_3_ nanoparticles (table 1). These values are larger than the respective particle diameters estimated from the HRTEM images because of the surface coating and hydration of the nanoparticles.

**Figure 4.**
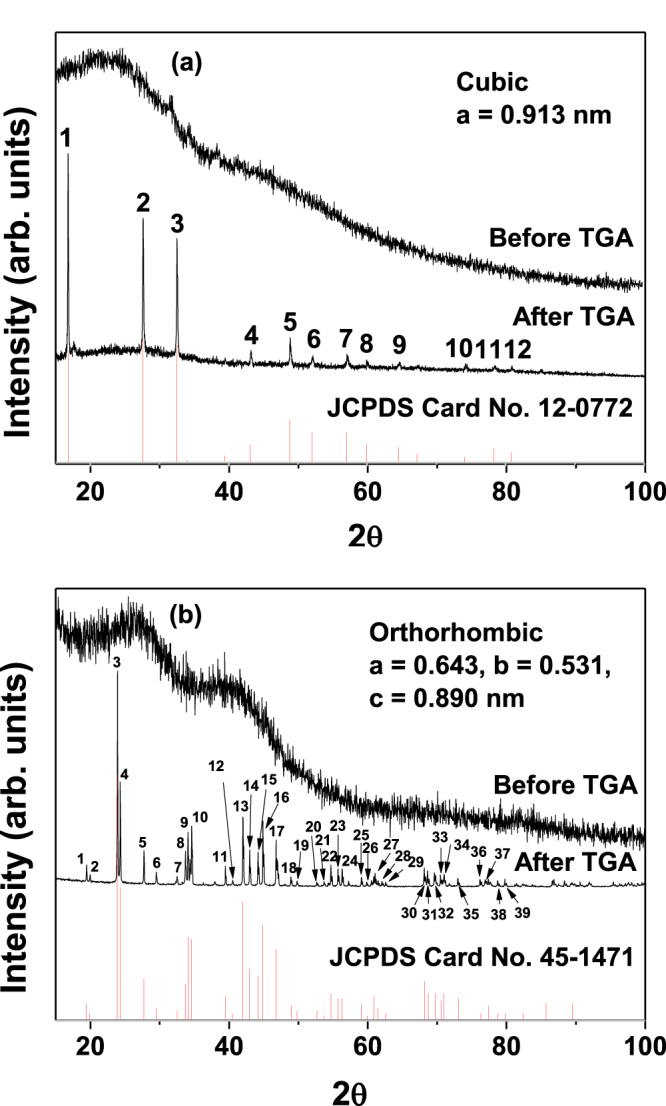
XRD patterns of powder samples of D-glucuronic acid coated (a) Na_2_WO_4_ and (b) BaCO_3_ nanoparticles: before (top) and after (middle) TGA. Reference XRD data from JCPDS are shown at the bottom [24, 25]. Full assignments of the peaks with (hkl) Miller indices are provided in the appendix.

The XRD patterns of the powder samples were recorded before and after TGA (figure 4), and it was found that the XRD patterns of all the samples before TGA were broad, due likely to the ultrasmall particle diameters and heavy surface coating with D-glucuronic acid [23]. Here, the TGA analysis was carried out to study chemical compositions of the as-synthesized nanoparticles through crystallization by TGA analysis. The XRD patterns of all the powder samples after TGA were sharp, indicative of high crystallinity in both samples. All the peaks after TGA could be assigned using the Miller indices (hkl) (appendix). Na_2_WO_4_ nanoparticles showed a cubic structure with an estimated cell constant of *a* = 9.13 Å (figure 4(a)), while BaCO_3_ nanoparticles showed an orthorhombic structure with estimated cell constants of *a* = 6.43, *b* = 5.31, and *c* = 8.90 Å (figure 4(b)). These values are consistent with previously reported values [24, 25].

### 3.2. Surface coating results

**Figure 5.**
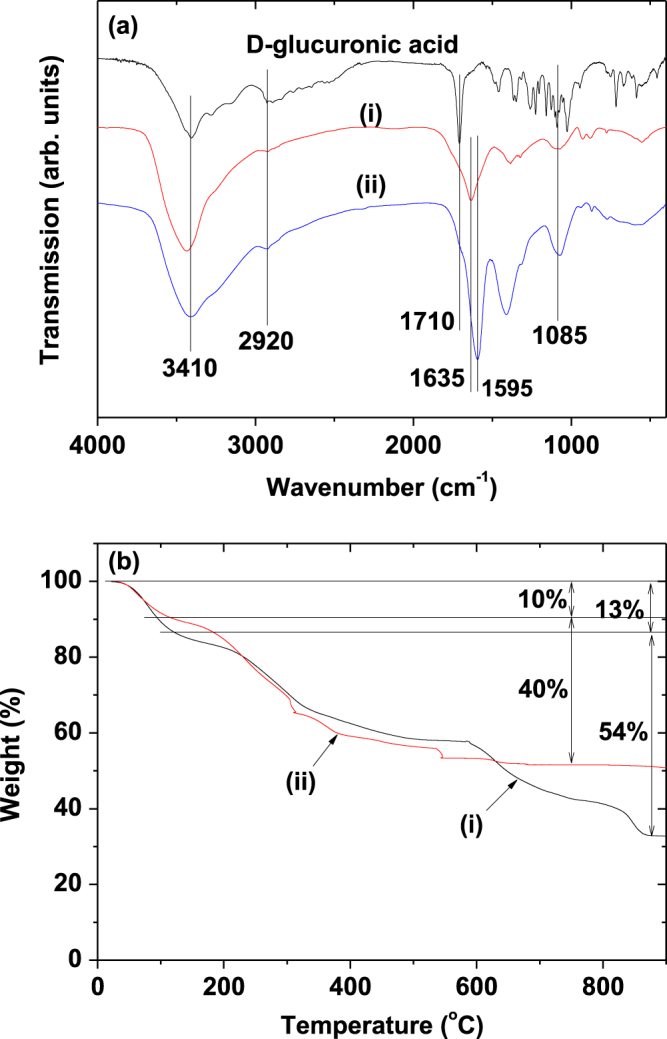
(a) FT-IR absorption spectra and (b) TGA curves of powder samples: D-glucuronic acid coated (i) Na_2_WO_4_ and (ii) BaCO_3_ nanoparticles.

Surface coating of the nanoparticles with D-glucuronic acid was investigated by recording FT-IR absorption spectra of the powder samples (figure 5(a)). The absorption peaks from D-glucuronic acid in the samples showed that the nanoparticles were successfully coated with D-glucuronic acid in all the samples: C–H stretch at 2930 cm^−1^, C=O stretch at 1595 and 1635 cm^−1^, and C–O stretch at 1085 cm^−1^ originating from D-glucuronic acid were observed. The D-glucuronic acid is bonded to a nanoparticle through its COOH functional group. This bonding corresponds to a hard acid (i.e., the nanoparticle)-hard base (i.e., the D-glucuronic acid)-type reaction [26–28]. The strongly bonded COOH showed red-shifts with respect to free COOH (figure 5(a)): 75 cm^−1^ for D-glucuronic acid coated Na_2_WO_4_ nanoparticles and 115 cm^−1^ for D-glucuronic acid coated BaCO_3_ nanoparticles. These red-shifts have been observed in many other cases [23, 29–32], supporting our results.

Sufficient surface coating of the nanoparticles with D-glucuronic acid crucial for water-solubility and biocompatibility are required for biomedical applications [33]. The amount of surface coating on each sample in this study was estimated as a weight percent (%; *P*) from the mass drop in the TGA curves generated (figure 5(b)), where an initial mass drop due to moisture desorption between room temperature and ∼105 °C was subtracted in estimating *P*. The grafting density (*σ*) [34], which corresponds to an average number of D-glucuronic acid molecules coated per unit surface area of a nanoparticle, was then estimated using the bulk density of the respective nanoparticles (4.179 g cm^−3^ for Na_2_WO_4_ and 4.43 g cm^−3^ for BaCO_3_) [35, 36], the *P* value estimated from the respective TGA curve, and the average particle diameter (*d*_avg_) estimated from the corresponding HRTEM image. By multiplying the estimated *σ* by the nanoparticle surface area 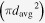 , the average number (*N*) of D-glucuronic acid molecules coated per nanoparticle was estimated 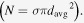 The estimated *P*, *σ*, and *N* values are shown in table 1. These large values indicate that sufficient surface coating was achieved for all samples.

### 3.3. *In vitro* cellular cytotoxicity results

**Figure 6.**
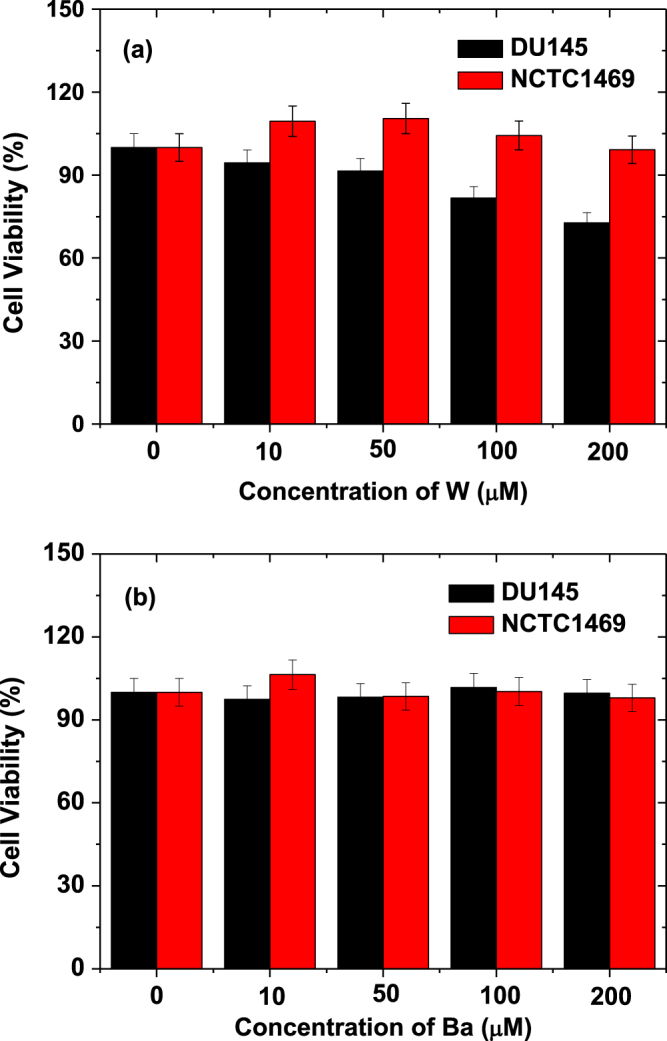
*In vitro* cell viabilities of aqueous sample suspensions: the treatment of NCTC1469 and DU145 cells with D-glucuronic acid coated (a) Na_2_WO_4_ and (b) BaCO_3_ nanoparticles.

Human prostate cancer (DU145) and normal mouse hepatocyte (NCTC1469) cells were used as test cells for cytotoxicity assessments. The cells were incubated with the aqueous sample suspensions for 48 h, and the cell viabilities of all the samples were found to be ≥90% following treatment with metal concentrations of up to 200 *μ*M (figures 6(a) and (b)). All samples are therefore considered biocompatible.

### 3.4. X-ray attenuation and phantom images

**Figure 7.**
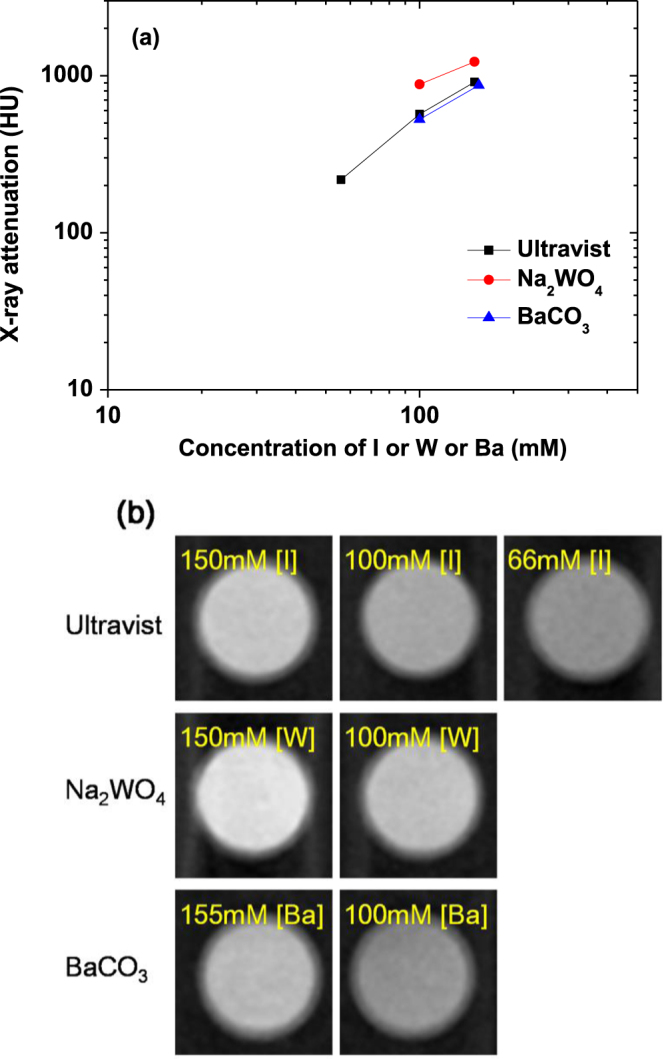
(a) X-ray attenuation and (b) x-ray attenuation phantom images at an x-ray source voltage of 70 keV for aqueous sample suspensions of D-glucuronic acid coated Na_2_WO_4_ and BaCO_3_ nanoparticles, and Ultravist^®^ as a function of the concentrations of [I] or [W] or [Ba]. Water was used as a reference.

The x-ray attenuation of aqueous sample suspensions was measured and compared to the commercial iodine CT contrast agent Ultravist^®^, which has three iodines per molecule with hydrophilic groups for water-solubility. X-ray attenuation depends on the voltage of x-ray source [16], and 70 keV was used in this study as in [19]. The x-ray attenuation was plotted as a function of [W] or [Ba] or [I] concentration (figure 7(a)). The observed x-ray attenuation of D-glucuronic acid coated Na_2_WO_4_ nanoparticles was found to be stronger than that of Ultravist^®^ at the same atomic concentration, whereas the x-ray attenuation of D-glucuronic acid coated BaCO_3_ nanoparticles was found to be slightly lower than that of Ultravist^®^. This can be explained by the fact that the linear x-ray attenuation coefficients can be ranked at W ≫ I ≈ Ba at an x-ray source voltage of 70 keV [13], (see footnote 7). These results imply that at the same number density, the x-ray attenuation of all the nanoparticle samples will be much stronger than those of iodine contrast agents because the nanoparticles contain large amount of metal per nanoparticle. X-ray attenuation phantom images were obtained (figure 7(b)), and it was found that the phantom images of an aqueous suspension of D-glucuronic acid coated Na_2_WO_4_ nanoparticles are brighter than those of Ultravist^®^, while the phantom images of an aqueous suspension of D-glucuronic acid coated BaCO_3_ nanoparticles are slightly darker than those of Ultravist^®^. These observations are consistent with the observed x-ray attenuation at the same atomic concentration (figure 7(a)).

### 3.5. Comparison between samples and Ultravist^®^

Based on the x-ray attenuation (figure 7(a)) and phantom image (figure 7(b)) data reported here, the CT capabilities of the samples and Ultravist^®^ can be ranked as Na_2_WO_4_ ≫ Ultravist^®^ ≈ BaCO_3_ at the same atomic concentration, which is consistent with the magnitude of the linear x-ray attenuation coefficients: W ≫ I ≈ Ba at an x-ray source voltage of 70 keV [13], (see footnote 7). However, at the same number density, all the nanoparticle samples will have a higher x-ray attenuation than Ultravist^®^ because the nanoparticles have a large number (*N*_metal_) of metal atoms per nanoparticle. Assuming spherical nanoparticles and using the *d*_avg_ estimated from the HRTEM images, *N*_metal_ was estimated to be ∼350 for the Na_2_WO_4_ nanoparticles and ∼360 for the BaCO_3_ nanoparticles by using a simple formula: *N*_metal_ ≈ (*x*/*y*)(*d*_avg_/*h*)^3^, where *x* = number of metal atoms per chemical formula, *y* = number of all the atoms per chemical formula, and *h* = average ionic diameter of all the atoms [37] in the chemical formula (0.23 nm for both Na_2_WO_4_ and BaCO_3_). Therefore, because Ultravist^®^ has three iodines per molecule, the atomic concentrations of the sample suspensions of D-glucuronic acid coated Na_2_WO_4_ and BaCO_3_ nanoparticles will be roughly 120*N*_d_ times higher than that of Ultravist^®^ at the same number density (*N*_d_). The x-ray attenuation of the sample suspensions of D-glucuronic acid coated Na_2_WO_4_ and BaCO_3_ nanoparticles will therefore in turn be roughly 120*N*_d_*A*_N_ (*A*_N_ = normalized x-ray attenuation of the sample suspensions relative to Ultravist^®^) times stronger than that of Ultravist^®^ at the same number density.

### 3.6. *In vivo* CT images

**Figure 8.**
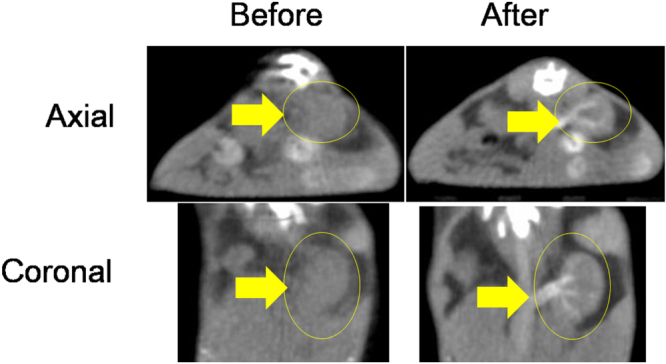
*In vivo* CT images of a mouse before (left) and after (right) intravenous injection of an aqueous sample suspension of D-glucuronic acid coated Na_2_WO_4_ nanoparticles into the tail vein: axial images (top) and coronal images (bottom). The kidney is labeled with an arrow and a circle.

D-glucuronic acid coated Na_2_WO_4_ nanoparticles have the highest x-ray attenuation power among the samples studied here including the Ultravist^®^, and an aqueous sample suspension of D-glucuronic acid coated Na_2_WO_4_ nanoparticles was therefore used to acquire *in vivo* CT images. Approximately 0.015 mmol W kg^−1^ (100 *μ*L of 150 mM [W]) was injected into an ICR mouse tail vein, and axial and coronal CT images were acquired before and after injection (figure 8). This injection dose is much lower than 2–6.4 mmol I kg^−1^, which has been typically used for iodine contrast agents in an ICR mouse [38]. Brighter contrast enhancements were clearly observed in the mouse kidney (labeled with arrow and circle) after injection, supporting the potential use of metal-containing nanoparticles as CT contrast agents. We plan to study higher concentrations for *in vivo* experiments as well as for cytotoxicity tests in the future.

## 4. Conclusions

Because of the strong x-ray attenuation of metals and the large amount of metal per nanoparticle in this study, these metal-containing nanoparticles represent potential CT contrast agents. To explore these potential agents, two different metal-containing nanoparticles (Na_2_WO_4_ and BaCO_3_) were synthesized and investigated for the first time. The results of the investigation are as follows.

(1) Water-soluble and biocompatible D-glucuronic acid coated Na_2_WO_4_ (*d*_avg_ = 3.2 ± 0.1 nm) and BaCO_3_ (*d*_avg_ = 2.8 ± 0.1 nm) nanoparticles were synthesized.
(2) D-glucuronic acid coated Na_2_WO_4_ nanoparticles exhibited a stronger x-ray attenuation than an iodine contrast agent at the same atomic concentration, whereas D-glucuronic acid coated BaCO_3_ nanoparticles were found to have a slightly weaker x-ray attenuation than the iodine contrast agent, owing to the magnitude of the corresponding linear x-ray attenuation coefficients: W ≫ I ≈ Ba at an x-ray source voltage of 70 keV. At the same number density, however, both nanoparticle samples have a much stronger x-ray attenuation than iodine contrast agents because the nanoparticles contain a large amount of metal per nanoparticle.
(3) *In vivo* CT images of a mouse were obtained after intravenous injection of an aqueous sample suspension of D-glucuronic acid coated Na_2_WO_4_ nanoparticles. Positive (or brighter) contrast enhancements in the CT images were clearly observed after injection, indicating that the metal-containing nanoparticles represent potential CT contrast agents.

## A1. Assignments of the peaks of Na_2_WO_4_ nanoparticles after TGA

(Peak number, hkl, 2*θ*): (1, 111, 16.74), (2, 220, 27.69), (3, 311, 32.52), (4, 331, 43.10), (5, 422, 48.77), (6, 511, 52.00), (7, 440, 57.10), (8, 531, 59.89), (9, 620, 64.53), (10, 551, 74.10), (11, 642, 78.36), (12, 731, 80.78).

## A2. Assignments of the peaks of BaCO_3_ nanoparticles after TGA

(Peak number, hkl, 2*θ*): (1, 011, 19.41), (2, 002, 19.97), (3, 111, 23.92), (4, 102, 24.32), (5, 200, 27.71), (6, 201, 29.52), (7, 210, 32.51), (8, 020, 33.71), (9, 211, 34.07), (10, 013, 34.62), (11, 022, 39.45), (12, 004, 40.57), (13, 122, 42.01), (14, 104, 42.98), (15, 220, 44.20), (16, 213, 44.94), (17, 311, 46.80), (18, 222, 48.92), (19, 204, 49.80), (20, 031, 52.68), (21, 024, 53.70), (22, 131, 54.70), (23, 124, 55.72), (24, 115, 56.34), (25, 322, 59.10), (26, 304, 59.86), (27, 231/033/411/402/224, 61.00), (28, 215, 62.05), (29, 006, 62.55), (30, 233, 68.12), (31, 413, 68.60), (32, 206, 69.70), (33, 324, 70.50), (34, 040/315, 71.00), (35, 026, 73.00), (36, 142, 76.19), (37, 511, 77.03), (38, 117, 78.70), (39, 226, 79.72).
